# Myeloid Cell Leukemia 1 and Hexokinase 2 Directly Interact to Form a Glucose Metabolic Regulatory Axis

**DOI:** 10.3390/cells15100891

**Published:** 2026-05-13

**Authors:** Robert Lee, Alexus Acton, Madeline Holliday, Nicholas J. Lennemann, William J. Placzek

**Affiliations:** 1 Department of Biochemistry and Molecular Genetics, The University of Alabama at Birmingham, Birmingham, AL 35294, USA; rlee3@uab.edu (R.L.);; 2 Department of Microbiology, The University of Alabama at Birmingham, Birmingham, AL 35294, USAnjlenn@uab.edu (N.J.L.)

**Keywords:** cancer, hexokinase 2 (HK2), MCL1, metabolism, metabolomics, NMR, non-small cell lung cancer (NSCLC)

## Abstract

**Highlights:**

**What are the main findings?**

- The anti-apoptotic protein, MCL1 directly binds to hexokinase 2 (HK2) resulting in an increase in enzymatic function.
- This is confirmed in a cellular context where MCL1 binding of HK2 enhances glucose metabolism.

**What are the implications of the main findings?**

- Provides a mechanistic basis for MCL1 impact on metabolism.
- Provides a key integration point between apoptotic regulators and cellular metabolic machinery.

**Abstract:**

Hexokinase 2 (HK2) catalyzes the first committed step of glucose metabolism—the conversion of glucose to glucose-6-phosphate—directing carbon flux into an array of metabolic pathways such as glycolysis, pentose phosphate pathways, amino acid biosynthesis, and others. Given its prominent role in glucose metabolism, it is critical we understand the regulation of HK2 to appreciate its role in normal physiological function as well as in disease states like cancers. In this study we sought to establish the ability of myeloid cell leukemia 1 (MCL1) to bind and regulate HK2 via its reverse Bcl-2 homology (rBH3) motifs. We employed a combination of biochemical and metabolic analysis in non-small cell lung cancer (NSCLC) cell models (H1299, A549, and NCI-H23) to establish a fundamental link between apoptosis and metabolic regulation. This demonstrates that MCL1 directly binds and enhances HK2 enzymatic activity through interactions with rBH3 on HK2. Consequently, we observe significant reductions in glucose-derived metabolites and impaired cellular metabolic plasticity with the disruption of the HK2-MCL1 interaction. These findings establish a novel mechanism by which anti-apoptotic proteins can directly regulate glucose metabolism.

## 1. Introduction

Control of cellular metabolism is essential for homeostasis, and its dysregulation is a hallmark of various disease states, especially cancers [1,2]. Cancers utilize dysregulated metabolism as both the energy source and building blocks for sustained anabolic processes necessary for rapid cell growth [3,4]. At the center of cellular metabolism is the hexokinase family of proteins. These proteins are responsible for the conversion of glucose to glucose-6-phosphate (G6P), trapping glucose in the cell and serving as feeder substrate for multiple downstream metabolic pathways [5]. While there are five hexokinases (HK1-4, and Hexokinase Domain Containing Protein 1 or HKDC1), it is HK2 that is primarily expressed in cancers and is implicated in driving the Warburg Effect [6,7,8,9]. Unsurprisingly, increased HK2 expression has been shown to increase tumor burden, promote proliferation, metastasis, and worsen prognosis [9,10,11].

Structurally, HK2 comprises two domains, the N- and C-terminal domains (NTD and CTD respectively) linked via a helical linker [12]. Both domains contain a catalytic core responsible for binding and coordinating glucose and ATP to form G6P [13]. Unlike other HK family members, HK2 utilizes both domains for catalysis, which enables it to have the fastest enzymatic rate [13]. This makes HK2 an ideal choice for rapidly growing cancerous cells [7]. Furthermore, these domains also serve as regulatory sites, containing: (1) mitochondrial localization sequences, important for aiding mitochondrial stability; (2) phosphorylation sites which regulate mitochondrial localization; (3) the allosteric feedback inhibition site of G6P [5,12,14,15,16]. Despite these advances in our understanding of HK2, little is known about direct protein regulators of HK2. Herein, we identify a novel regulatory mechanism of HK2 through a direct binding interaction with myeloid cell leukemia-1 (MCL1) which enhances HK2 catalysis. MCL1 is a pro-survival member of the B-Cell lymphoma 2 (Bcl-2) protein family [17]. Canonically MCL1 prevents apoptosis by binding and sequestering pro-death Bcl-2 homology 3 (BH3)-containing proteins (e.g., BAK, BAX, BIM, etc.) [18,19].

We have previously reported that MCL1 binds a novel reversal of the canonical BH3 (rBH3) motif, mediating non-canonical functions beyond the Bcl-2 family including participation in transcriptional regulation (via p73), RNA metabolism (via PTBP1), and cell cycle progression (via CDKN2C) [20,21,22,23]. Despite the reversal of the native BH3 motif, the rBH3 maintains key consensus hydrophobic and acidic residues with the native BH3 motif, preserving MCL1’s binding capacity [20]. Like these interacting partners, HK2 has also been found to contain rBH3 motifs in the NTD and CTD, providing a novel mechanism by which MCL1 can regulate HK2’s catalytic function. While prior work demonstrated that MCL1 and HK2 form a complex at the mitochondrial membrane with a Voltage Dependent Anion Channel (VDAC), the molecular regulatory mechanism remained unsolved [24]. Here we sought to demonstrate that MCL1 binding to HK2 via NTD and CTD localized rBH3 motifs alters HK2 enzymatic turnover to provide a direct molecular mechanism for this observation and to ultimately link apoptosis to glucose metabolism.

## 2. Materials and Methods

### 2.1. Cell Culture

H1299 and A549 cells were maintained in a humidified environment with 5% CO_2_ in 1 g/L glucose DMEM (Corning Life Sciences, Durham, NC, USA), supplemented with 10% FBS (Corning Life Sciences, Durham, NC, USA) and 100 units/mL each of penicillin and streptomycin, and 0.25 µg/mL of Fungizone antimycotic (Life Technologies, Grand Island, NY, USA). U2OS and NCI-H23 cells were passaged in RPMI 1640 (Corning Life Sciences, Durham, NC, USA), supplemented with 10% FBS (Corning Life Sciences, Durham, NC, USA) and 100 units/mL each of penicillin and streptomycin, and 0.25 µg/mL of Fungizone antimycotic. Cell lines were obtained from ATCC (Manassas, VA, USA) and validated with short tandem repeat analysis at the Heflin Center Genomics Core Facility at UAB.

### 2.2. Recombinant Protein Purification

Using the New England BioLabs transformation protocol, MCL1, SUMO1, HK2, and D209A/D657A HK2 constructs were transformed into BL21 (DE3) *E. coli*. The HK2 construct used was a gift from Cheryl Arrowsmith (Addgene plasmid # 25529). Competent bacteria were grown in 500 mL cultures (250 mL per flask) with kanamycin selection at 37 °C until an optical density (OD600) of 0.5–0.7 was reached. At this point protein expression was induced with 1 mM final concentration Isopropyl-β-D-1thiogalactopyranoside (IPTG, Fisher Bioreagents, Pittsburgh, PA, USA). For MCL1 and SUMO1, OD_600_ optical density was measured for several hours until a plateau was reached. For HK2 constructs, after induction, flasks were placed in an 18 °C incubator for 18 h. After, for all constructs, cells were harvested via centrifugation at 4700× *g*. Pellets obtained were frozen at −80 °C for future purification. For purification, pellets were resuspended in 20 mL of protein lysis buffer (MCL1 and SUMO-50 mM HEPES and 150 mM NaCl at pH 7.4; HK2 constructs–100 mM Tris-HCl, 150 mM NaCl, 5 mM Imidazole, 3 mM β-mercaptoethanol (BME) at pH 7.4). Once resuspended, the lysate was supplemented with two EDTA-free mini protease inhibitor tablets (Pierce Chemical Co., Dallas, TX, USA, A32955) and lysozyme (0.25 mg/mL). Lysis was achieved via probe sonication for 7 min on ice. Cell debris was subsequently pelleted at 14,000× *g* and supernatant was filtered through a 0.45 µm filter (Millex). Protein was purified on a BioRad NGC FPLC (BioRad Laboratories, Hercules, CA, USA) system using nickel chromatography (1 mL HisTrap, GE Healthcare, Chicago, IL, USA) followed by gel filtration. For MCL1 and SUMO1 a 16/60 Sephacryl S-100 (GE Healthcare, Chicago, IL, USA) column was used. For HK2 constructs a 16/60 Sephacryl S-200 (GE Healthcare, Chicago, IL, USA) column was used. SDS-PAGE analysis was used to confirm protein identity and purity. All protein constructs were validated with Sanger Sequencing. HK2 constructs were stored in a final buffer of 50 mM HEPES, 150 mM NaCl, 3 mM BME, pH 7.4 and frozen at −80 °C until ready for use. MCL1 and SUMO were stored in a final buffer of 50 mM HEPES, 150 mM NaCl pH 7.4 at 4 °C until ready for use.

### 2.3. Competitive FPA

**Figure 1:**
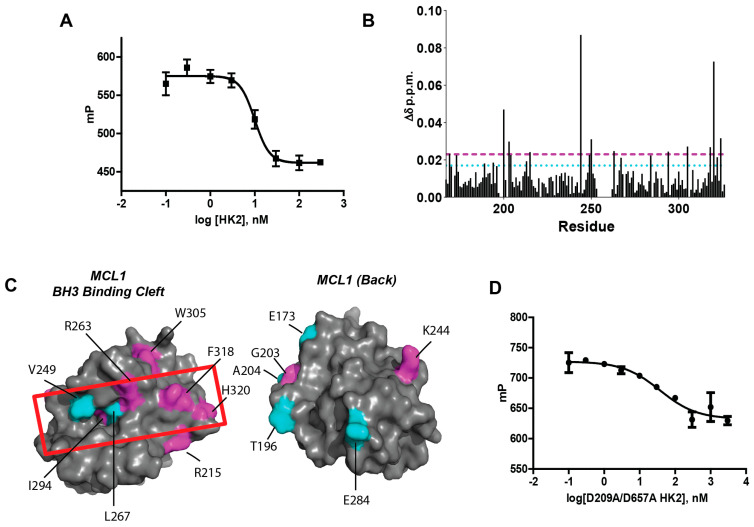
MCL1 Directly Binds HK2 via the rBH3 Motif. (**A**) Competitive FPA consisting of 100 nM recombinant MCL1, 10 nM F-BAK and increasing concentrations of recombinant HK2. (**B**) Chemical shift perturbation analysis of 2D [^1^H−^15^N]-TROSY HSQC spectra of 35 µM ^15^N-MCL1 + 10% D_2_O or 35 µM ^15^N-MCL1 + 38.5 µM HK2 + 10% D_2_O. Chemical shift perturbation (CSP) was calculated using the equation Δδ ppm = √(ΔδH^2^ + (ΔδN/5)^2^) for each amino acid of MCL1. The cyan line indicates a CSP of greater than 1 standard of deviation (SD) from the mean, the magenta line represents a CSP of greater than 2 SD of the mean. (**C**) Mapped chemical shift perturbations on a space filled model of MCL1. The left represents the front view of MCL1 where a majority of CSPs fell within the BH3 binding cleft of MCL1 (highlighted in the red box). The right represents the 180° rotation of MCL1 where minimal CSP was appreciated. Cyan residues represent 1 SD CSP, magenta represents 2 SD CSP. (**D**) Competitive FPA consisting of 100 nM recombinant MCL1, 10 nM F-BAK and increasing concentrations of recombinant rBH3 mutated D209A/D657A HK2.

80 µL of titrated recombinant HK2 (100 pM to 300 nM final for HK2, 100 pM to 3 µM final for D209A/D657A HK2) was added to a black untreated 96-well microplate (ThermoScientific, Waltham, MA, USA) with 10 µL 10× MCL1 (final concentration 100 nM) [21]. These proteins were incubated for 20 min and shaken at 300 rpm to allow for binding. Subsequently, 10 µL of 10× F-BAK (final concentration 10 nM) was added per well. The plate was then covered with an opaque lid and allowed to shake for an additional hour. After incubation, the plate was read using the FP-Fluorescein setting (1.0 s, CW lamp filter-F485, emission filter, F535) on a Perkin Elmer Victor X5 plate reader (Perkin Elmer, Shelton, CT, USA). The buffer used for all assays was 50 mM HEPES, 150 mM NaCl, pH 7.4. All experiments were completed in technical and biological triplicate. All data plotted in Figure 1 is one representative assay of a technical triplicate.

### 2.4. Split GFP Cloning

MCL1-GFP_1–10_ was constructed through the amplification of MCL1 plasmid using MCL1-GFP_1–10_ forward and reverse primers. Amplified products were ligated into the BamHI linearized pcDNA3.1-GFP_1–10_-HiFi vector via HiFi Assembly. HK2-GFP_11_ was generated through the amplification of a FLAG-HK2 plasmid using HK2-GFP_11_ primers. Amplified products were ligated into the BamHI linearized pcDNA3.1-GFP_11_-HiFi vector via HiFi Assembly. GFP-HiFi vectors were generated through the insertion of GFP_1–10_ or GFP_11_ between HindIII and BamHI restriction sites of pcDNA3.1. All ligations were transformed into DH5α *E. coli*. Indicated constructs were (co)-transfected in U2OS cells with polyethyleneimine (PEI) at a 1:1 ratio of DNA (µg) to 1 mg/mL PEI stock (µL). For a complete list of primers see Supplemental Table S1.

### 2.5. Immunoprecipitation (IP)

H1299 cells were seeded at 2.5 × 10^5^ cells in a 6-well plate 24 h prior to transfections. Two micrograms of pcDNA3.1 plasmid DNA (containing FLAG vector or FLAG-HK2) was mixed with OptiMEM, P3000, and L3000 for 5 min at room temperature. Next, 1 g/L glucose DMEM media +2.5% FBS was aspirated off and replaced with fresh media. After incubation the transfection reagent was added onto the cells and placed into the incubator for 48 h. Cells were harvested via trypsin dissociation. Cell lysates were lysed in Peirce IP Buffer and supplemented with 1× Halt Protease Inhibitor Cocktail with EDTA (ThermoScientific, Waltham, MA, USA). Then, 250 µg of cell lysates as quantified by the Bicinchoninic acid assay (BCA) were incubated with 5 µg of recombinant MCL1 for 1 h at 4 °C. After this, 20 µL of anti-FLAG magnetic beads were added to each sample along with BSA (0.1% final) and incubated rotating at 4 °C for 1 h. Immune complexes were washed three times with Peirce IP buffer (Pierce Chemical Co., Dallas, TX, USA). Each sample was denatured from the magnetic beads with 4× Laemmli sample buffer containing BME and boiled at 95 °C for 10 min. Proteins were analyzed via Western blot as described below.

### 2.6. Western Blot Analysis:

Cells were collected via trypsin dissociation and lysed in 1× RIPA lysis buffer supplemented with 1× Halt Protease Inhibitor Cocktail with EDTA (ThermoScientific, Waltham, MA, USA). Sample amounts were quantified using BCA. Each sample was denatured with 4× Laemmli sample buffer (ThermoScientific, Waltham, MA, USA) containing BME at 95 °C for 10 min. 20 ng of lysates were run on a SDS polyacrylamide gel electrophoresis using 4–15% Mini-PROTEAN TGX Stain-Free Gels (Bio-Rad, Hercules, CA, USA) at 150 V for 45 min and transferred to a PVDF membrane using a TransBlot Turbo semi-dry transfer system at 25 V and 1.3 A for 7 min. Membranes were blocked in 5% *w*/*v* nonfat milk in phosphate-buffered saline + 0.01% tween (PBST) for 1 h. Primary antibodies were incubated at 4 °C overnight. Blots were then washed with PBST. Secondary antibodies were incubated for 1 h at room temperature and washed with PBST. All Western blots were visualized with ECL reagent (Bio-Rad, Hercules, CA, USA) and imaged on a Bio-Rad ChemiDoc MP imaging system (Bio-Rad, Hercules, CA, USA).

### 2.7. Primary Antibodies

All primary antibodies were diluted 1:1000 in 1% milk-PBST. HK2: anti-HK2 rabbit mAb (C64G5, CST); MCL1: anti-MCL1 rabbit mAb (D2W9E, CST); FLAG: anti-FLAG rabbit mAb (D6W5B, CST).

### 2.8. Secondary Antibodies

Secondary antibodies were diluted in 1% milk to 1:2000. Rabbit: goat anti-rabbit IgG-HRP (CST).

### 2.9. HK2 Enzyme Kinetics

HK2 reaction rate was measured spectroscopically by coupling the reaction of HK2 to glucose-6-phosphate dehydrogenase (G6PDH). The rate of HK2 was monitored via the rate of NADPH production (ε_340_ = 6220 M^−1^cm^−1^) using a Perkin Elmer Victor X5 plate reader (CW lamp filter F-485, absorbance filter F-340). Enzymatic reactions were performed at 25 °C in 50 mM HEPES, 150 mM NaCl (pH 7.4), 20 mM MgCl_2_, 3 mM NADP^+^, and 2.5 µU/µL G6PDH (Fisher Scientific, Waltham, MA, USA) at different glucose (0.03 mM–2 mM) and ATP (0.075 mM–4 mM) concentrations in untreated 96-well plates. MCL1 and SUMO titrations to the reaction were performed at various concentration ratios from 1:1 protein:HK2 to 10:1 protein:HK2. All data were plotted in Microsoft Excel to determine initial enzyme velocity. Each biological replicate’s reaction rate represents an average rate obtained from a technical duplicate. Michaelis–Menten parameters were calculated using Prism GraphPad v.8 enzyme kinetics module. Error bars were calculated for biological triplicates of each reaction.

### 2.10. MTS Assay Kit for Cell Dose–Response

Cells were seeded in a 96-well plate in RPMI 1640 (1×) (-Phenol Red) (Corning Life Sciences, Durham, NC, USA) with 2.5% FBS. Cells were placed into a humidified atmosphere of 5% CO_2_ at 37 °C for 8 h and then dosed on a 9-point dose–response curve with a final DMSO concentration of 0.25% in both experimental and control samples. After 72 h, cells were treated with 10 µL of MTS assay reagent (ab197010) and incubated at 37 °C for 1 h. The 96-well plate was read on a PerkinElmer Victor X5 Multimode Plate Reader (Perkin Elmer, Shelton, CT, USA) at 490 nm. Cell dose responses can be found in Supplemental Figure S1A–C.

### 2.11. Nuclear Magnetic Resonance (NMR)

2D [^1^H−^15^N]-TROSY-HSQC spectra were acquired using a Bruker 850 MHz magnet (Bruker BioSpin, Billerica, MA, USA) at the Central Alabama High Field NMR Facility. Samples were prepared on the day of collection with 35 µM ^15^N-MCL1 or 35 µM ^15^N-MCL1:38.5 µM HK2 (1:1.1) in 50 mM HEPES, 150 mM NaCl, pH 7.4, supplemented with sodium azide, TCEP (Fisher Bioreagents, Pittsburgh, PA, USA) and deuterium oxide (99%, Cambridge Isotope Laboratories, Inc., Tewksbury, MA, USA). Spectra were analyzed using Computer-Aided Resonance Assignment (CARA v1.8.4.2). Peak lists generated in CARA were exported to Microsoft Excel for the CSP quantification, and the calculation of mean and standard of deviation. CSP were calculated using the formula: √(ΔδH^2^ + (ΔδN/5)^2^) [25]. Any peaks exhibiting a significant CSP (≥1 SD) were mapped to the space filled model of MCL1 in PyMOL v2.6 using 2PQK. The BIM peptide was removed to display the BH3 binding pocket.

Metabolomics NMR data were collected using a Bruker 850 MHz magnet at the Central Alabama High Field NMR facility. Samples prepared from metabolite extraction were resuspended in 210 µL of 100 mM Na_2_HPO_4_, 0.02% sodium azide, and 500 µM DSS in deuterium oxide. Then, 200 µL of resuspension were added to a 3 mm NMR tube and spectra were collected immediately. NOESYGPPR1D (^1^H-NMR) and zgpg30 (^13^C-NMR) were collected for each sample. HSQCETGPSI (2D [^1^H−^13^C]-HSQC) were collected on select control samples for further confirmation of metabolite peak identity. Spectra were initially processed in Bruker Topspin (Bruker BioSpin, Billerica, MA, USA) before exporting to Chenomx NMR Suite v.8 (Chenomx, Inc., Edmonton, Alberta, Canada) for the quantification and identification of metabolites. ^13^C-NMR metabolite abundance was calculated by integrating the area under the curve (A.U.C) of a metabolite’s peak, which is directly proportional to the concentration of the metabolite. These calculations were performed in Bruker Topspin v3.2 (Bruker BioSpin, Billerica, MA, USA) and normalized to viable cell count. All metabolites were identified using a combination of Human Metabolome Database (HMDB) reference spectra, Biological Magnetic Resonance Bank (BMRB), 2D [^1^H−^13^C]-HSQC NMR, and 850 MHz reference databases present in Chenomx NMR Suite. Example spectra with labeled metabolites and chemical shifts can be found in Supplemental Figures S2 and S3 and Supplemental Table S2. A schematic of ^13^C incorporation into downstream glucose metabolites can be found in Supplemental Figure S4.

### 2.12. Metabolite Preparation and Harvest

H1299 cells were seeded in 5.5 mM glucose (Fisher Scientific), 2.05 mM L-glutamine (Gibco, Grand Island, NY, USA) with 2.5% dialyzed FBS (Corning Life Sciences, Durham, NC, USA) DMEM (Gibco, Grand Island, NY, USA) in a treated 10 cm dish. NCI-H23 cells were seeded in 5.5 mM glucose (Fisher Scientific, Waltham, MA, USA), 2.05 mM L-glutamine (Gibco, Grand Island, NY, USA), 1 mM sodium pyruvate (Gibco, Grand Island, NY, USA) with 2.5% dialyzed FBS (Corning Life Sciences, Durham, NC, USA) DMEM (Gibco, Grand Island, NY, USA) in a treated 10 cm dish. Sodium pyruvate was added to aid with the cell adherence of NCI-H23 cells. After 2 days, cells were washed with 10 mL DPBS followed by changing media to 5.5 mM [U−^13^C] glucose (99%, Cambridge Isotope Laboratories, Inc., Tweksbury, MA, USA), and 2.05 mM L-glutamine with 2.5% dialyzed FBS. At this point cells were treated with MCL1 inhibitor S63845 dissolved in DMSO or DMSO (control) at a final concentration of 0.25% for both groups and maintained for 24 h. During harvest, cell media was removed, and cells were washed with ice cold DPBS (10 mL). DPBS was removed and cells were quenched with ice cold 80:20 methanol:molecular grade water (5 mL) and scraped. Plates were subsequently washed with an additional 2 mL of methanol:molecular grade water and placed in dark conical tubes. The metabolite slurry was vortexed for 15 s followed by 15 min incubation on ice. Then, 5 mL of ice-cold chloroform was added to the aqueous solution and vortexed for 15 s followed by 15 min incubation on ice. Samples were centrifuged at 14,000× *g* for 15 min at 4 °C. The top aqueous layer was subsequently removed. 5 mL of ice-cold molecular grade water was added to the samples prior to being snap-frozen in liquid nitrogen. Samples were subsequently lyophilized at −86 °C before NMR sample preparation. After the acquisition of ^1^H-NMR, samples were snap-frozen and lyophilized to maintain sample integrity prior to the resuspension and collection of ^13^C-NMR. Matched samples were used to collect trypan blue cell viability counts.

### 2.13. Cellular Proliferation Curves

H1299 and A549 cells were seeded in 5.5 mM glucose, 2.05 mM L-glutamine with 2.5% dialyzed FBS DMEM in a treated 6-well dish and grown in a humidified environment with 5% CO_2_. For non-glucose depleted growth curves, cells were allowed to adhere for 6 h followed by treatment with either 0.25% DMSO (vehicular control) or S63845. Cell counts were subsequently collected every 24 h for 3 days via trypsin dissociation and washed with DPBS. Viability and counts were assessed using a Bio-Rad Automated Cell Counter (Bio-Rad Laboratories, Hercules, CA, USA) with equal parts cells and trypan blue. For glucose depleted curves, cells were seeded as above and allowed to adhere for 18 h. Following this time, cell media was removed, cells were washed with DPBS and 2.05 mM L-glutamine with 2.5% dialyzed FBS DMEM was added to the cells for 6 h. On completion of this time, cells were washed and supplemented with 5.5 mM glucose, 2.05 mM L-glutamine with 2.5% dialyzed FBS DMEM and either vehicular control, vehicular control with 5 mM pyruvic acid, S63845, or matched concentration S63845 with 5 mM pyruvic acid. Cell counts and viability were repeated as above for 3 days.

### 2.14. Reagents

S63845 (Chemietek, Indianapolis, IN, USA): MCL1 inhibitor. All other reagents described above.

### 2.15. Statistical Analysis

All experiments were performed in biological triplicates except H1299 DMSO controls for ^13^C-NMR which were biological duplicates due to the loss of one sample during the second lyophilization step. Data is expressed as mean ± SD. Differences between two data sets were calculated using the two-tailed unpaired Student’s *t*-test, with *p* < 0.05 considered statistically significant. Differences between three or more data sets were calculated using one-way ANOVA with Tukey’s Post Hoc testing, with *p* < 0.05 considered statistically significant. Statistical analyses were performed in Prism v 8 (GraphPad Inc., La Jolla, CA, USA) or Microsoft Excel. * *p* < 0.05, ** *p* < 0.01, *** *p* < 0.001.

## 3. Results

### 3.1. MCL1-HK2 Binding Occurs via the BH3-Binding Cleft

We previously established MCL1 binds to, and modulates, protein function through a naturally occurring rBH3 motif found in other cellular proteins (p73, CDKN2C, and PTBP1) [21,22,23]. BLAST (blast.ncbi.nlm.nih.gov/blast.cgi, accessed on 28 April 2026) sequence analysis of the human proteome revealed HK2 contains putative rBH3 motifs within both its NTD and CTD (Table 1). Strikingly, key acidic residues of the rBH3 for HK2 D209 and D657—which we demonstrated to be important for MCL1 binding—are contained within the catalytic cores of HK2 [12]. These aspartic acid residues are essential for the coordination of the 6′-hydroxyl group of glucose with the γ-phosphate of ATP [26]. Alanine substitution at these positions renders HK2 catalytically inactive despite preserving tertiary structure [13].

We previously demonstrated MCL1 binds other rBH3 containing proteins (p73 and PTBP1) through its BH3-binding cleft [20,21,22,23]. To determine whether HK2 similarly targets the BH3-binding cleft, we performed a competitive fluorescence polarization assay (FPA) with recombinant HK2 and MCL1 [27]. Following established methods, we incubated MCL1 with increasing concentrations of HK2 [21,23]. Subsequently we added 10 nM of a FITC-labeled BAK peptide (F-BAK)—a native ligand of MCL1—to determine HK2’s ability to outcompete F-BAK [21]. HK2 displaced F-BAK from the BH3-binding cleft of MCL1 with a KD of less than 10 nM (below the limit of detection), confirming a high-affinity binding interaction (Figure 1A).

We then conducted ^1^H−^15^N-TROSY HSQC using matched ^15^N-labeled MCL1 samples with and without HK2 (ratio HK2:MCL1—1.1:1) to identify binding residues. In brief, a comparative analysis of mapped amide peaks of MCL1 before and after the addition of HK2 revealed significant changes in the chemical environment (e.g., chemical shift perturbation) for select amide peaks of MCL1 (Supplemental Figure S5) [28]. Chemical shift perturbation was calculated using δΔppm shifts in an iterative approach to determine the significantly shifted residues (defined as ≥1σ δΔppm shift) (Figure 1B) [29]. Mapping these significantly shifted residues onto the crystal structure of MCL1 (2PQK) revealed that most residues were clustered with the BH3-binding cleft of MCL1 (ex. V249, M250, R263, I294, F318, H320), which is consistent with other rBH3-mediated interactions (Figure 1B,C). Notably, R263—which forms a critical salt bridge in BH3 motif binding—was also perturbed by HK2, mirroring other rBH3 proteins [21,23,29].

### 3.2. The rBH3 Motifs Mediate HK2-MCL1 Binding

Having localized the HK2-MCL1 binding to the BH3 cleft, we next investigated if rBH3 motifs of HK2 mediate the binding interaction. Prior studies of rBH3 proteins demonstrated that the alanine substitution of the acidic residue of the rBH3 motif significantly weakens binding [21,22,23]. Thus, we generated double rBH3 mutant D209A/D657A HK2 and assessed the binding interaction with FPAs as before. D209A/D657A HK2 significantly weakened the MCL1 binding affinity (KD = 44.2 ± 10.6 nM) compared to WT-HK2 (KD ≤ 10 nM), confirming rBH3 motifs are essential for interaction (Figure 1D).

### 3.3. HK2 and MCL1 Bind in a Cellular Context

**Figure 2:**
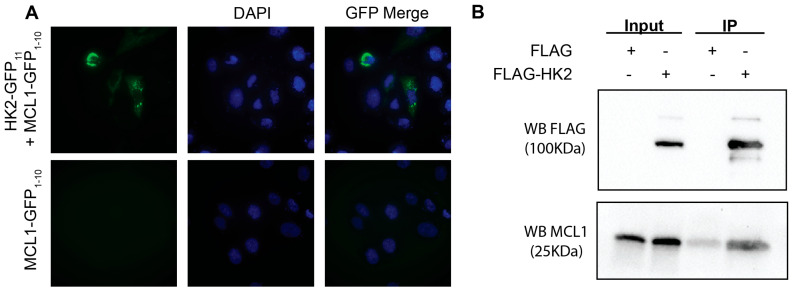
MCL1 Binds HK2 in a cellular context. (**A**) Representative images of U2OS cells transfected with either MCL1-GFP_1–10_ alone or MCL1-GFP_1–10_ + HK2-GFP_11_. Immunofluorescence was analyzed on an Olympus IX83 (Center Valley, PA, USA) at 60× magnification and analyzed in ImageJ v 1.54 for protein localization. (**B**) The co-immunoprecipitation (Co-IP) of FLAG-HK2 or FLAG control transiently transfected H1299 cells. Co-IP was performed with anti-FLAG magnetic beads. Western blot analysis was used to analyze FLAG tagged HK2 construct protein expression and the pulldown of recombinant MCL1.

Having confirmed binding in an in vitro context, we investigated if HK2 and MCL1 bind in cellulo. First, we verified the intracellular proximity between HK2 and MCL1 through a split GFP reporter system [30]. We tagged MCL1 with a GFP_1–10_ tag (MCL1-GFP_1–10_) and HK2 with a GFP_11_ peptide (HK2-GFP_11_) to assess proximity. As previously described when a GFP_1–10_ protein and a GFP11 peptide are within 15 Å, the holo-GFP protein is formed, and fluorescence is observed [30]. The transient transfection of U2OS cells with MCL1-GFP_1–10_ alone resulted in no observable fluorescence while co-transfection with HK2-GFP_11_ yielded cytoplasmic fluorescence, confirming that MCL1 and HK2 co-localize within a distance compatible with direct binding (Figure 2A). To validate HK2-MCL1 binding, we employed co-immunoprecipitation (Co-IP). We transiently transfected non-small cell lung cancer (NSCLC) H1299 cells with FLAG-HK2 or FLAG control for 48 h prior to lysate collection. We analyzed MCL1 protein presence through Co-IP western blot. We observed that FLAG-HK2 robustly pulled down MCL1, confirming the interaction between the proteins in a cellular context (Figure 2B).

### 3.4. MCL1 Enhances HK2 Catalytic Activity

**Figure 3:**
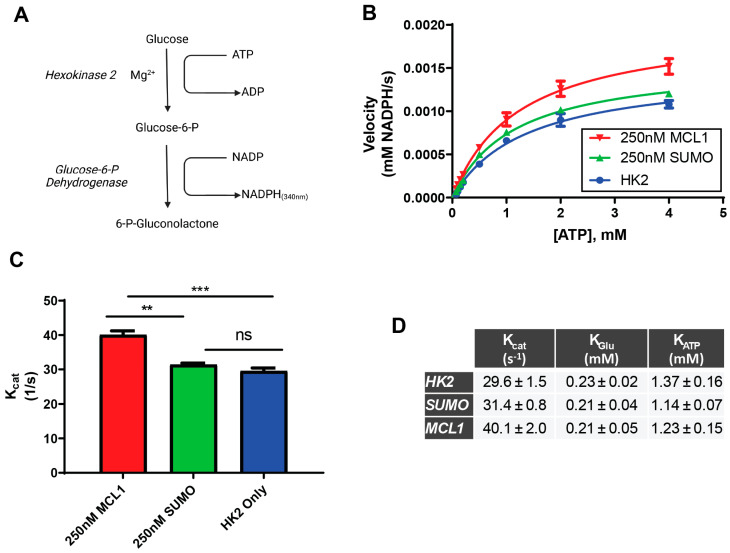
MCL1 Positively Increases HK2 Enzymatic Turnover Without Impacting Substrate Affinity. (**A**) Schematic of the coupled assay used in Michaelis–Menten enzymatic analysis of HK2. Created with Biorender (www.biorender.com). (**B**) Plot of the velocity of HK2 at varying concentrations of ATP with a fixed concentration of glucose with and without 250 nM MCL1 or 250 nM SUMO. Each point represents a triplicate of experimental data, and the line is the best fit calculation for a Michaelis–Menten Plot. (**C**) One-way ANOVA statistical analysis on the calculated K_cat_ values generated from Figure 2B. (**D**) Summary of Michaelis–Menten parameters obtained for HK2 only, HK2 + 250 nM MCL1 and HK2 + 250 nM SUMO. Each value and error were calculated from an average triplicate ATP and glucose Michaelis–Menten plots. ** *p* < 0.01, *** *p* < 0.001, ns—not statistically significant (*p* > 0.05).

Since the rBH3 motifs mediating HK2-MCL1 binding are contained within HK2’s catalytic core, we investigated the functional consequences of this interaction. Using established Michaelis–Menten enzyme kinetics assays, we monitored HK2’s activity by coupling G6P production with NADPH via G6PDH (Figure 3A) [31,32]. We titrated MCL1 against a fixed concentration of HK2 which revealed a striking increase in catalysis. We observed a 35.1 ± 2.5% (*p* = 0.0003) increase in HK2 turnover (K_cat_) when compared to HK2 alone (MCL1:HK2 = 5:1) without impacting substrate affinity (K_M_) (Figure 3B–D).

To rule out any non-specific protein interactions driving this effect, we titrated matched concentrations of small ubiquitin-like modifier 1 (SUMO) against HK2. We chose SUMO as a negative control because it has not been characterized to bind HK2. Under matched SUMO concentrations (SUMO:HK2 = 5:1) we observed an insignificant change in K_cat_ (6.2 ± 0.4%, *p* = 0.3589) or K_M_ when compared to HK2 only, though higher SUMO concentrations induced artificial changes (Supplemental Figure S6A). Notably, the D209A/D657A mutant HK2 could not be analyzed due to a lack of catalytic activity, as expected (Supplemental Figure S6B) [13]. These results demonstrate how MCL1 binding potentiates HK2 activity despite engaging with residues which are critical for catalysis [33].

### 3.5. MCL1 and HK2 Form a Glucose Metabolic Regulatory Axis

**Figure 4:**
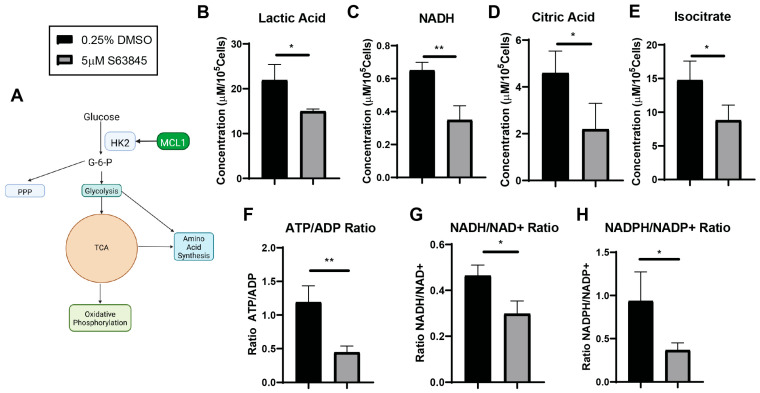
^1^H-NMR Reveals Decreased Metabolites, Cellular Energetics and Redox Metabolites. (**A**) Simplified schematic of how HK2 feeds into glycolysis, TCA, oxidative phosphorylation, PPP, and amino acid biosynthesis. (**B**–**E**) Student’s *t*-test summary of the indicated metabolites harvested from H1299 cells; graphs were generated from Table 2. Metabolites showed significant decreases in S63845 treated groups when compared to DMSO vehicular control. Each metabolite and error are representative of a triplicate. (**F**–**H**). Student’s *t*-test on the ratio calculation of energetic and redox metabolites from Table 2 as indicated. Average and error bars represent a triplicate for each ratio. (**A**) created with Biorender. * *p* < 0.05, ** *p* < 0.01.

Having established that MCL1 binding enhances HK2 catalysis in vitro, we investigated next if this effect carried into a cellular context. To assess the impact of this interaction and its subsequent impact on cellular glucose metabolism, we utilized the NSCLC cell lines H1299 and NCI-H23 that both have documented dependence on MCL1 and HK2 [34]. We treated HK2/MCL1 expressing NSCLC cell lines H1299 and NCI-H23 with either the BH3 binding cleft inhibitor S63845 (which we previously identified blocks rBH3 interactions) or vehicular control (Figure 4A) [22,23,34,35,36,37]. We then used nuclear magnetic resonance (NMR) metabolomics to assess glucose metabolism. Based on our in vitro enzymatic studies, we hypothesized that MCL1 inhibition would decrease HK2-driven metabolism. We assessed a variety of glucose and non-glucose related metabolites using ^1^H-NMR in H1299 cells (Table 2). We observed significant reductions in HK2 associated metabolites in glycolysis (lactic acid, NADH); citric acid cycle (TCA) (NADH, citric acid, isocitrate); oxidative phosphorylation (ATP/ADP ratio); and pentose phosphate pathways (PPP) (NADPH/NADP^+^ ratio) (Figure 4B–H). Notably, despite being often derived from G6P, non-essential amino acids (alanine, glycine, glutamine, glutamic acid and serine) showed no significant reduction in levels of ^1^H-NMR (Supplemental Table S3). We observed similar patterns of metabolic change in NCI-H23 cells (Supplemental Figure S7A,B).

To confirm that the metabolic changes observed were associated with HK2 activity rather than a global reduction in metabolism, we analyzed non-glucose associated metabolites across multiple pathways. We assessed phenylalanine metabolism (tyrosine), essential amino acids metabolism (valine, isoleucine, leucine, and phenylalanine), and amino acid and lipid related metabolism (methylmalonic acid, o-phosphocholine) which remained unchanged (Table 2, Supplemental Table S3). This ruled out non-specific metabolic suppression. Trypan blue viability assays confirmed that cell viability remained greater than 93%, ruling out decreased cell viability as the cause of the metabolic changes observed (Supplemental Figure S8A–D).

**Figure 5:**
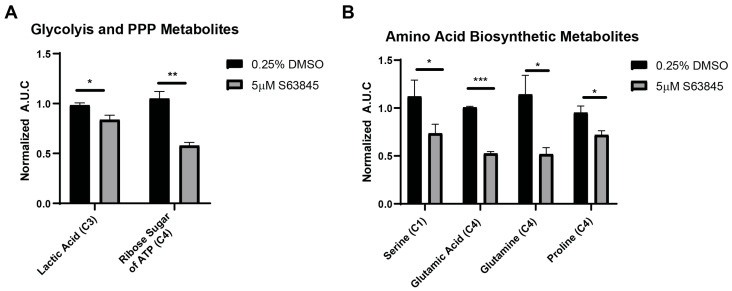
[U−^13^C] Glucose Tracer Analysis Confirms ^1^H-NMR Findings and Reveals Decreased Amino Acid Biosynthesis. (**A**,**B**) ^13^C metabolite analysis of 5 µM S63845 treatment in H1299 cells. ^13^C-NMR was collected in 100 mM Na_2_HPO_4_ + 0.02% NaN_3_ + 500 µM DSS in D_2_O. Concentrations of indicated metabolites were quantified by integrating the area under the curve (A.U.C) of ^13^C peak intensity measurements, as calculated in Bruker Topspin. A.U.C was normalized to viable cell count. Each plot represents a mean and error for the indicated metabolite. * *p* < 0.05, ** *p* < 0.01, *** *p* < 0.001.

To confirm HK2-mediated glucose metabolism was the cause of the observed metabolic changes, we conducted ^13^C-NMR tracer studies using the [U−^13^C] glucose we supplemented the cell’s media with. As hypothesized, we observed significant reductions in the concentrations of ^13^C labeled lactic acid (C3) and PPP-related ATP ribose sugar (C4) compared to vehicular controls (Figure 5A). Furthermore, while not showing significant changes in ^1^H-NMR, ^13^C-NMR showed significantly impaired non-essential amino acid biosynthesis as shown by decreases in the concentrations of ^13^C in amino acids serine (C1), glutamic acid (C4), glutamine (C4), and proline (C4) (Figure 5B). This could be due to the enhanced specificity of ^13^C glucose tracers, showing glucose’s contribution to amino acid biosynthesis versus all metabolic pathway contributions (such as glutamine or lipid metabolism) to the overall pool of non-essential amino acids as in ^1^H data [38].

Our 1H-NMR and ^13^C-NMR tracer studies confirm that MCL1 inhibition significantly reduces glucose metabolism as shown by a reduction in metabolites in all major glucose-utilizing pathways [39]. These findings together are consistent with the impairment of HK2 catalysis upon MCL1 dissociation as shown by the reduction in glucose derived metabolites, the unchanged non-glucose associated metabolites, and the preserved cell viability.

### 3.6. The MCL1-HK2 Axis Mediates Metabolic Plasticity Under Glucose Stress

Our metabolomics findings established MCL1-HK2 comprise a glucose metabolic regulatory axis. To investigate its functional consequences, we assessed cellular proliferation. We initially treated HK2 and MCL1 expressing H1299 and A549 cells with S63845, a BH3 mimetic that ablates rBH3 binding, or vehicular control in 1 g/L glucose containing DMEM media, and measured proliferation over a 3-day period with trypan blue [35,36,37]. Consistent with prior studies in NSCLC, proliferation was not impacted (Supplemental Figure S9A–D) [40].

**Figure 6:**
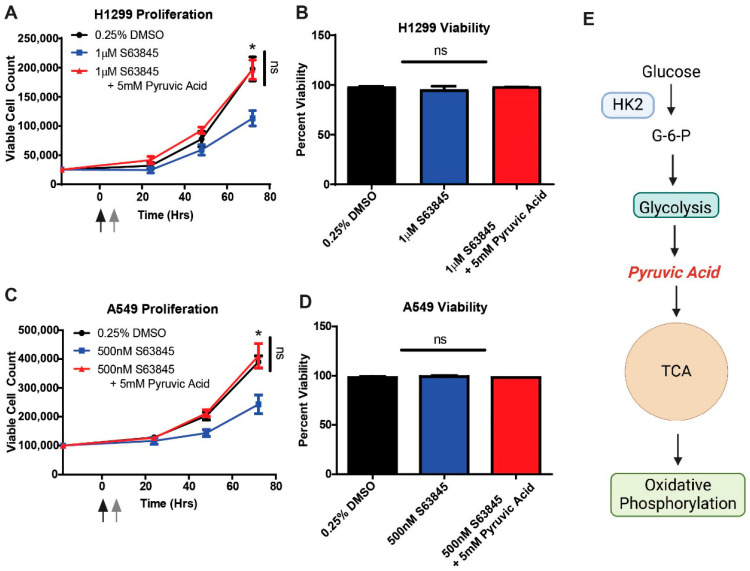
Ablation of MCL1/HK2 Cellular Binding Results in Decreased Metabolic Plasticity. (**A**) Proliferation curves of H1299 cells treated with vehicular control, 1 µM MCL1 inhibitor S63845 or 1 µM S63845 in media supplemented with 5 mM pyruvic acid to rescue impaired HK2-driven glycolysis. Black arrow represents start of glucose depletion to simulate glucose metabolic stress (6 h). Gray arrow represents reintroduction of glucose as well as treatment with indicated group. (**B**) Viability and cell count of H1299 cells on day 3 after treatment as assessed by trypan blue. (**C**) Proliferation curves of A549 cells treated with vehicular control, 500 nM S63845 or 500 nM S63845 in media supplemented with 5 mM pyruvic acid. Black arrow represents start of glucose depletion to simulate glucose metabolic stress (6 h). Gray arrow represents reintroduction of glucose as well as treatment with indicated group. (**D**) Viability and cell count of A549 cells on day 3 after treatment as assessed by trypan blue. Error bars were calculated for biological triplicate. (**E**) Schematic of how pyruvic acid rescues impaired HK2 glycolysis. (**E**) created with Biorender. * *p* < 0.05, ns—not statistically significant (*p* > 0.05).

Strikingly, when cells were subjected to glucose stress, as simulated by glucose deprivation for 6 h prior to S63845 treatment, we observed a 42.8 ± 11.7% reduction in proliferation (*p* = 0.030) in H1299 cells with no impact on viability (Figure 6A,B). To mechanistically link this phenotype to HK2 impairment, we employed a metabolic rescue. The supplementation of media with 5 mM pyruvic acid (which bypasses HK2-dependent glycolysis) restored proliferation in S63845 treated cells (−0.7% ± 0.2%, *p* = 0.998 versus vehicular control) (Figure 6A,E) [41]. Importantly, pyruvic acid treatment did not significantly impact proliferation in control cells, demonstrating the specificity of this rescue (Supplemental Figure S10A–D). These trends were reflected in the A549 cell line as well (Figure 6C,D). These data demonstrate that the HK2-MCL1 axis is essential for metabolic adaptation to glucose stress.

## 4. Discussion

This work establishes a novel, non-canonical function for the pro-survival protein MCL1 as a direct regulator of HK2 through an rBH3-mediated interaction. Through binding FPA studies and NMR chemical shift perturbation analysis, we localized binding to the BH3-binding cleft of MCL1. Mutational analysis of HK2 localized the binding interaction to its rBH3 motifs. We demonstrated that the molecular consequence of binding is a 35.1% increase in HK2 turnover (K_cat_). This was carried into a cellular metabolic phenotype, where we observed significantly impaired glucose metabolism, aligning with an impairment in HK2 function. Finally, we observed that a functional consequence of the HK2-MCL1 axis is a reduced glucose metabolic plasticity as observed by a significant reduction in proliferation under glucose stress. More broadly, this rBH3-mediated interaction represents a key integration point between cell survival mechanisms and metabolic regulation.

Our findings also reveal a surprising functional consequence of HK2-MCL1 interaction. We hypothesized that MCL1 binding to HK2 would result in the occlusion of an active site as MCL1’s size would likely obstruct substrate access to at least one domain’s essential catalytic residues (D209 and/or D657). However, despite this, we observed a significant increase in HK2 activity. This presents an intriguing question: how mechanistically does MCL1 enact this effect? There are multiple (non-exclusive) hypotheses by which this could occur including: (1) allosteric activation of HK2, whereby MCL1 induces a confirmation change in the opposite catalytic site; (2) same-sided activation whereby MCL1 optimizes activity directly in the bound catalytic site; or (3) MCL1 binding relieves substrate inhibition. Substrate inhibition relief could not be assessed due to the coupled nature of the assay used. Further mutational analysis of HK2 and a HK2-MCL1 complex structure may help answer these questions.

Our work, in the context of others, also sheds light on how MCL1 carefully regulates bioenergetics. By binding HK2, MCL1 enhances glycolytic flux while simultaneously enhancing oxidative phosphorylation downstream by binding ATP synthase as demonstrated by Perciavalle et al. [42]. Thus, MCL1 inhibition creates synergistic metabolic stress whereby upstream glycolytic and TCA flux are significantly decreased and coupled with significant reductions in ATP synthesis potential [43]. This dual mechanism could be particularly devastating to cancers, whose reliance on glucose (e.g., Warburg Effect) and increased bioenergetic demands make them vulnerable to such coordinated impairment [44,45]. Bioenergetic crisis or “metabolic catastrophe” may heighten the effect of MCL1 inhibitors in highly glycolytic tumors that are dependent on MCL1 survival [43]. A limitation of this study is that it was only performed in cellular models, and it will be interesting to see how this is carried forward into in vivo systems.

Our metabolic rescue studies with pyruvic acid supplementation also reveal a potential mode of resistance to MCL1 inhibitors. Prior work by the DeBerardinis group observed heterogeneous metabolism in NSCLC tumors whereby the hypoxic core of the tumor relies more on glycolytic metabolism, while lactic acid-driven oxidative metabolism sustains the tumor edge [46,47,48]. Since lactic acid can be readily converted to pyruvic acid via lactic acid dehydrogenase (LDH), our data suggests that tumors could exploit this pathway to bypass HK2 impairment brought by MCL1 inhibition [39]. It would be interesting to see if this mechanistically occurs in acquired resistance cell models and if so, if this could be reversed using an LDH inhibitor.

Similarly, the MCL1-HK2 regulatory axis likely extends beyond cancers and plays a key role in insulin sensitive tissue (e.g., cardiomyocytes, muscle cells) homeostasis. As such, disruption of the HK2-MCL1 axis may provide further insight into MCL1 inhibitors’ recent struggles in clinical trials given the key role that both MCL1 and HK2 serve in cardiac metabolism and survival [49,50,51,52]. Going forward this opens exciting opportunities to explore this axis utilization in both healthy tissue function and disease tissues, like cancers, which are a result of, or rely on dysregulated metabolic and/or apoptotic function.

## 5. Conclusions

In this study, we establish a regulatory link between apoptotic machinery and glucose metabolism. Using biochemical and cellular approaches, we demonstrate that MCL1 directly binds to hexokinase 2 (HK2), leading to increased enzymatic turnover of HK2. In cells, this interaction enhances glucose metabolism, as shown by metabolomic analysis comparing conditions with and without the inhibition of MCL1 binding to HK2. Furthermore, loss of the HK2-MCL1 axis can be rescued through supplementation with pyruvic acid, a downstream product of glycolysis, which bypasses the axis. This rescue further confirms that the observed metabolic effects are dependent on HK2.

## Figures and Tables

**Table 1:** HK2 Contains a Putative rBH3 motif. Sequence alignment between the canonical BH3 motif, previously characterized reverse BH3 (rBH3) motifs, and motif containing proteins and HK2’s putative rBH3 motifs contained within the NTD and CTD of the protein. Conserved residues are colored with the critical L/M and D/E residues highlighted in red and hydrophobic residues colored blue. Phi represents a generic hydrophobic residue.

BH3 motif:	X Φ XXX L XX Φ G D X Φ
BAK_BH3_:	GQ V GRQ L AI I G D D I NRRYD
rBH3-1:	HLYAQMLEVTEN-NH_2_
rBH3-2:	YYYTLMTNVTEN-NH_2_
p73_rBH3_:	PQPVLEMLELSEKLKM-NH_2_
P18_rBH3_:	GAGNAQMLSVVENRGY-NH_2_
PTBP1_rBH3_:	STYYNVMTNAAEETNM-NH_2_
HK2_rBH3-NTD_:	DYGCTMMTGVTDNVVA-NH_2_
HK2_rBH3-CTD_:	EFGCTMMTGVTDNVVA-NH_2_

**Table 2:** ^1^H-NMR Reveals Decreased Metabolites in Pathways Downstream of HK2. Glucose derived metabolite abundance changes with 5 µM S63845 treatment in H1299 cells. On day 2 cells were given fresh media and treated with 5 µM S63845 or vehicular control and grown for 24 h. Metabolites were extracted, and ^1^H-NMR was collected in 100 mM Na_2_HPO_4_ + 0.02% NaN_3_ + 500 µM DSS in D_2_O. Metabolites were quantified in Chenomx. Metabolite concentrations were normalized to viable cell count. * *p* < 0.05, ** *p* < 0.01, ns—not statistically significant (*p* > 0.05).

Pathway	Metabolite	DMSO [µM/10^5^ Cells]	S63845 [µM/10^5^ Cells]	Percent Difference	*p*-Value	
Glycolysis	Lactic Acid	21.92 ± 3.49	14.25 ± 1.01	−35.02%	0.027	*
NAD+/NADH	NAD+	1.41 ± 0.15	1.16 ± 0.09	−17.49%	0.066	ns
	NADH	0.65 ± 0.05	0.35 ± 0.09	−46.33%	0.006	**
TCA	Citric Acid	4.59 ± 0.94	2.19 ± 1.10	−52.24%	0.046	*
	Isocitrate	14.75 ± 2.84	8.81 ± 2.25	−40.25%	0.047	*
	Malic Acid	13.55 ± 5.76	11.43 ± 5.15	−15.67%	0.659	ns
Ox-Phos	ADP	1.09 ± 0.31	2.42 ± 0.08	121.24%	0.002	**
	ATP	1.26 ± 0.08	1.08 ± 0.22	−14.27%	0.260	ns
PPP	NADP+	0.24 ± 0.05	0.39 ± 0.07	60.32%	0.039	*
	NADPH	0.22 ± 0.03	0.14 ± 0.01	−35.90	0.016	*
Non-Glucose Derived Metabolites	Valine	19.58 ± 6.58	20.44 ± 4.44	4.36%	0.861	ns
	Tyrosine	6.44 ± 2.23	7.19 ± 1.33	11.69%	0.642	ns
	Methylmalonic Acid	15.68 ± 2.21	19.26 ± 1.75	22.83%	0.092	ns
	Phosphocholine	35.70 ± 6.33	30.08 ± 2.51	−15.75%	0.226	ns

## Data Availability

The original contributions presented in this study are included in the article/Supplementary Material. Further inquiries can be directed to the corresponding author.

## Supplementary Materials

The following supporting information can be downloaded at: https://www.mdpi.com/article/10.3390/cells15100891/s1, Table S1: Primers Used in Split GFP Cloning. Figure S1: MTS Assay 3 Day Dose–Response Curves of Cell Lines Treated with S63845. Figure S2: ^1^H-NMR Spectra with Labels Detailing Position of Metabolites. Table S2: NMR Table Detailing Chemical Shift Information Used to Identify and Quantify Analyzed Metabolites. Figure S3: Representative ^13^C-NMR Spectra and Table of Chemical Shift Information. Figure S4: ^13^C Label Incorporation Scheme. Figure S5: Overlayed 2D [^1^H−^15^N]-TROSY HSQC of MCL1 and MCL1 + HK2. Figure S6: Summary of Michaelis–Menten Kinetics for HK2. Table S3: ^1^H-NMR Metabolites from Non-Essential and Other Non-Glucose Derived Metabolites. Figure S7: NCI-H23 Cell Metabolomics Reveal Similar Patterns of Metabolic Perturbation as H1299 Cells. Figure S8: Cell Counts and Viability of Cells Used for Metabolomics Analysis. Figure S9: S63845 Does Not Significantly Impact Proliferation Without Glucose Deprivation. Figure S10: 5 mM Pyruvic Acid Treatment Does Not Significantly Impact Proliferation.

## Abbreviations

The following abbreviations are used in this manuscript:

A.U.C	Area Under the Curve
BH3	Bcl-2 Homology 3
CSP	Chemical Shift Perturbation
CTD	C-Terminal Domain
F-BAK	FITC-labeled BAK Peptide
FPA	Fluorescence Polarization Assay
G6P	Glucose-6-Phosphate
HK2	Hexokinase 2
MCL1	Myeloid Cell Leukemia 1
NSCLC	Non-small Cell Lung Cancer
NTD	N-Terminal Domain
PPP	Pentose Phosphate Pathway
rBH3	Reverse Bcl-2 Homology 3
SUMO	Small Ubiquitin-like Modifier 1
TCA	The Citric Acid Cycle
