# Supplementary material for: Myeloid Cell Leukemia 1 and Hexokinase 2 Directly Interact to Form a Glucose Metabolic Regulatory Axis
Source: Cells. 2026 May 13;15(10):891. doi: 10.3390/cells15100891 (PMC13204606; doi:10.3390/cells15100891)
Supplement: Supplementary file 1 [file cells-15-00891-s001.zip › cells-4267396-supplementary.pdf]

Primers (5'-3')	
**MCL1-GFP$_{1-10}$ Forward**	AGTCCAGTGTGGTGGAATTCGGCGGCggtggttcaggaTCTAAGGGCGAAGAACTGTTC
**MCL1-GFP$_{1-10}$ Reverse**	ACGGGCCCTCTAGACTCGAGTCACTTTTCATTCGGATCTTTTGA
**HK2-Forward**	TTGGTACCGAGCTCGGATCCatgattgcctcgcatctgcttgcctac
**HK2-Reverse**	TTCCACCACACTGGAtcgctgtccagcctcacggatg

Supplemental Table S1. Primers Used in Split GFP Cloning.

Table contains the sequences of the primers used to clone MCL1-GFP$_{1-10}$ and HK2-GFP$_{11}$.

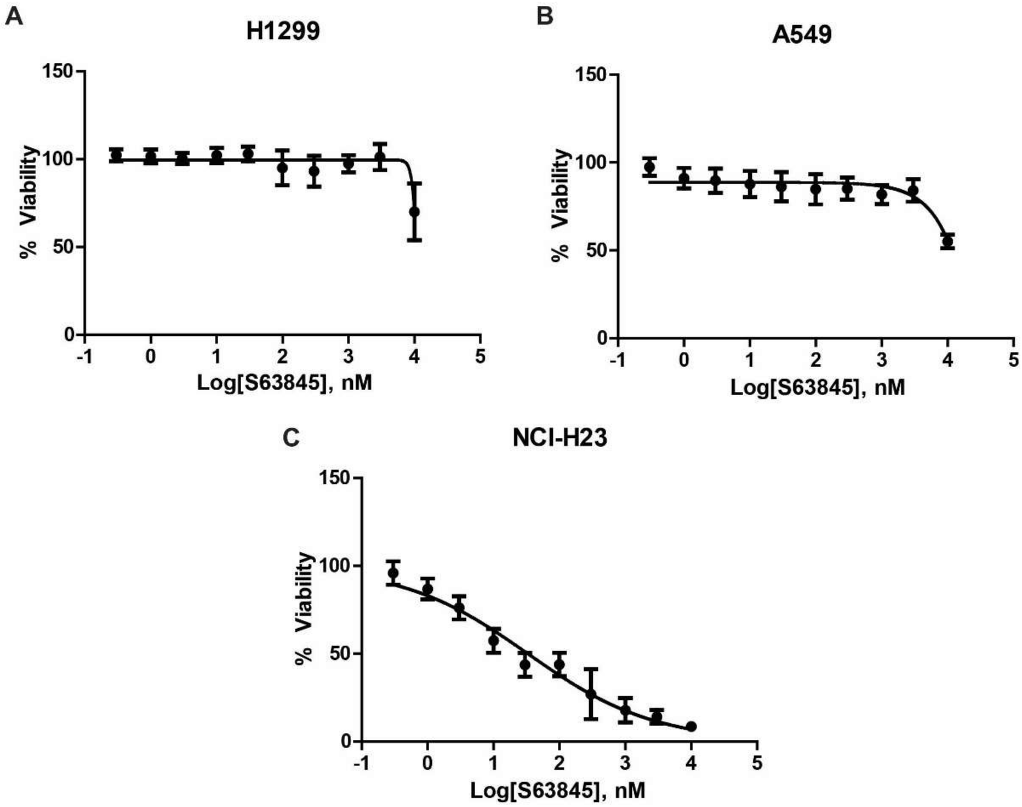

Supplemental Figure S1. MTS Assay 3 Day Dose Response Curves of Cell Lines Treated with S63845.

A) H1299 cell dose response curve. B) A549 cell dose response curve. C) NCI-H23 cell dose response curve. Cells were allowed to adhere for 8hrs and then dosed on a 9-point dose–response curve with a final DMSO concentration of 0.25%. After 72hrs, cells were treated with 10µL of MTS assay reagent (ab197010) for 1hr. Error bars were calculated for biological triplicate.

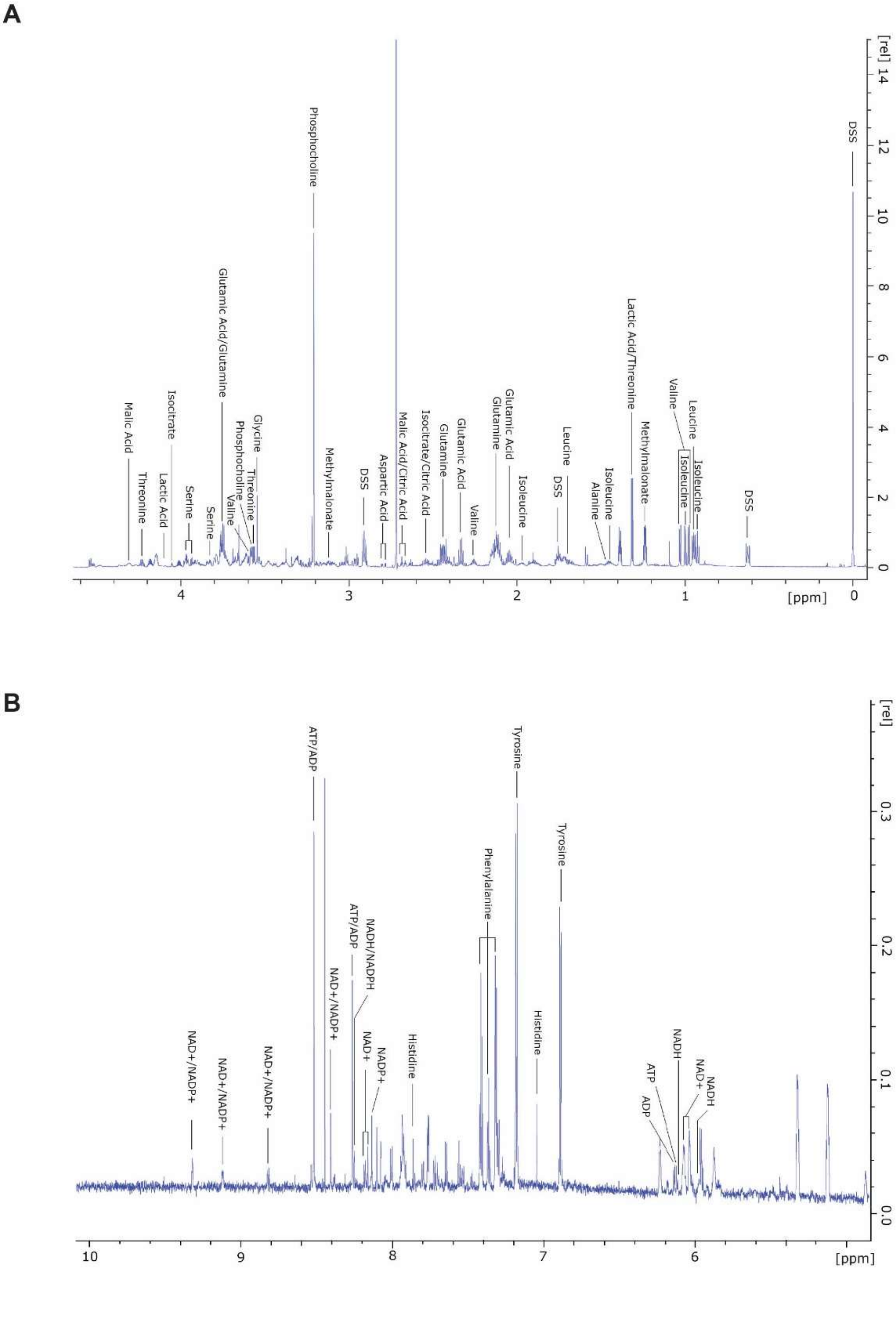
A
B
[rel]
[ppm]
DSS
Phosphocholine
Glutamic Acid/Glutamine
Malic Acid
Threonine
Isocitrate
Lactic Acid
Serine
Valine
Glycine
Threonine
Methylmalonate
Aspartic Acid
Malic Acid/Citric Acid
Isocitrate/Citric Acid
Glutamine
Glutamic Acid
Isoleucine
Leucine
Alanine
Lactic Acid/Threonine
ATP/ADP
NADH/NADPH
NAD+/NADP+
NAD+
NADP+
Histidine
Phenylalanine
Tyrosine
ATP
ADP
NADH

Supplemental Figure S2. ^{1}H-NMR Spectra with Labels Detailing Position of Metabolites.

A) Representative ^{1}H-NMR spectra of the aliphatic region from the H1299 DMSO control group with labelled chemical shift locations of analyzed metabolites. B) Representative ^{1}H-NMR spectra of the aromatic region from the H1299 DMSO control group with labelled chemical shift locations of analyzed metabolites.

Metabolite	Chemical Shifts (ppm) Used for Identification and Quantification
ADP	8.518 (s), 8.258 (s), 6.140 (d)
ATP	8.518 (s), 8.263 (s), 6.127 (d)
Alanine	1.469 (d)
Aspartic Acid	2.796 (m), 3.885 (m)
Citric Acid	2.524 (d), 2.678 (d)
Glutamic Acid	2.043 (td) 2.322 (t), 2.348 (t)
Glutamine	2.422 (m), 2.457 (m)
Glycine	3.547 (s)
Isocitric Acid	2.556 (m), 2.967 (m), 4.055 (d)
Isoleucine	0.927 (t), 0.998 (d), 1.457 (m), 1.970 (m)
Lactic Acid	1.315 (d), 4.100 (q)
Leucine	0.944 (t), 0.954 (m), 1.673 (m)
Malic Acid	2.396 (dd), 2.676 (dd)
Methylmalonic Acid	3.171 (q)
NAD+	9.322 (s), 9.12 (d), 8.822 (d), 8.409 (s), 8.180 (t), 8.162 (s), 6.078 (d)
NADH	8.447 (s), 8.251 (s), 7.046 (s), 6.118 (d), 5.965 (d)
NADP+	9.323 (s), 9.120 (d), 8.821 (d), 8.407 (s), 8.178 (s), 8.136 (s)
NADPH	8.460 (s), 8.250 (s), 6.183 (d)
O-Phosphocholine	3.210 (s), 3.581 (t), 4.150 (m)
Phenylalanine	7.417 (m), 7.366 (m), 7.319 (m)
Serine	3.824 (dd), 3.932 (m)
Threonine	1.315 (d), 3.565 (d), 4.233 (m)
Tyrosine	7.182 (d), 6.890 (d)
Valine	0.979 (d), 1.030 (d), 2.261 (m), 2.598 (d)

Supplemental Table S2. ^{1}H-NMR Table Detailing Chemical Shift Information Used to Identify and Quantify Analyzed Metabolites.

Table contains chemical shift information of the indicated metabolite along with multiplicity. Each of these peaks were used for identification and quantification of the indicated metabolite.

A

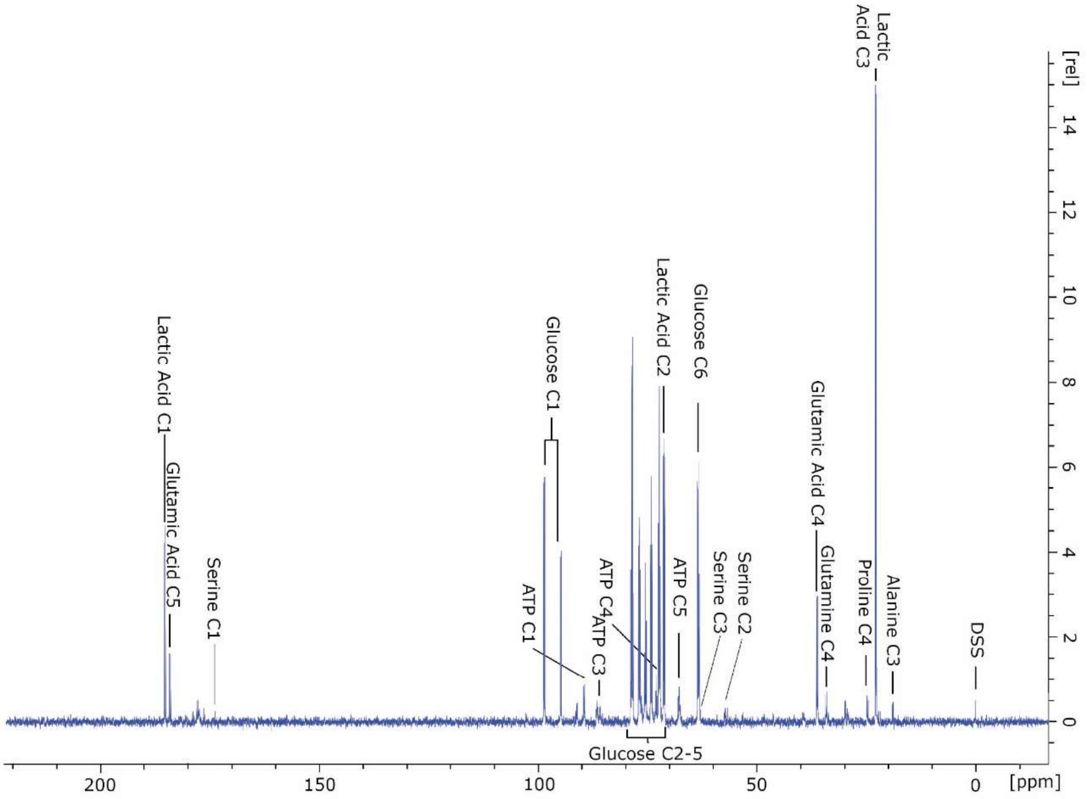

B

Chemical Shift List		
Metabolite	Carbon	Shift (ppm)
Alanine	C3	18.93
Lactic Acid	C1	185.24
	C2	71.17
	C3	22.78
Proline	C4	24.69
Glutamine	C4	34.12
Glutamic Acid	C4	36.16
	C5	184.08
Serine	C1	173.83
	C2	56.99
	C3	62.90
Ribose Sugar of ATP	C1	89.47
	C3	86.55
	C4	72.88
	C5	67.72
Glucose	C1	98.58, 94.76
	C2<br>C3<br>C4<br>C5	72.27,<br>74.14,<br>75.45,<br>76.79, 78.52
	C6	63.33

Supplemental Figure S3. Representative ^{13}C-NMR Spectra and Table of Chemical Shift Information.

A) Representative ^{13}C-NMR spectra from the H1299 DMSO control group with labelled chemical shift locations of analyzed metabolites. B) ^{13}C-NMR table detailing chemical shift information used to quantify and identify metabolites assessed. Chemical shift information represents the center of a multiplet.

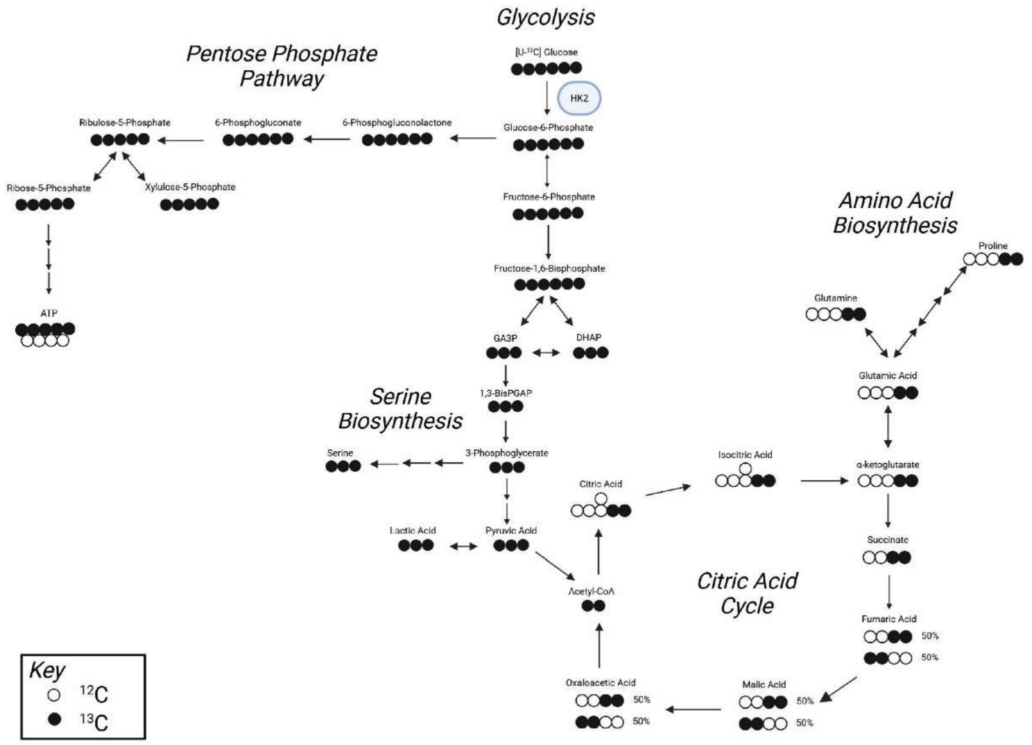

Supplemental Figure S4. ^{13}C Label Incorporation Scheme.

Schematic detailing how [U-^{13}C]-glucose incorporates into downstream metabolites. Carbon numbering is left to right in ascending order. White dots represent ^{12}C carbon, black dots represent ^{13}C carbon incorporation. TCA cycle represents a single complete cycle turn. Created with Biorender.

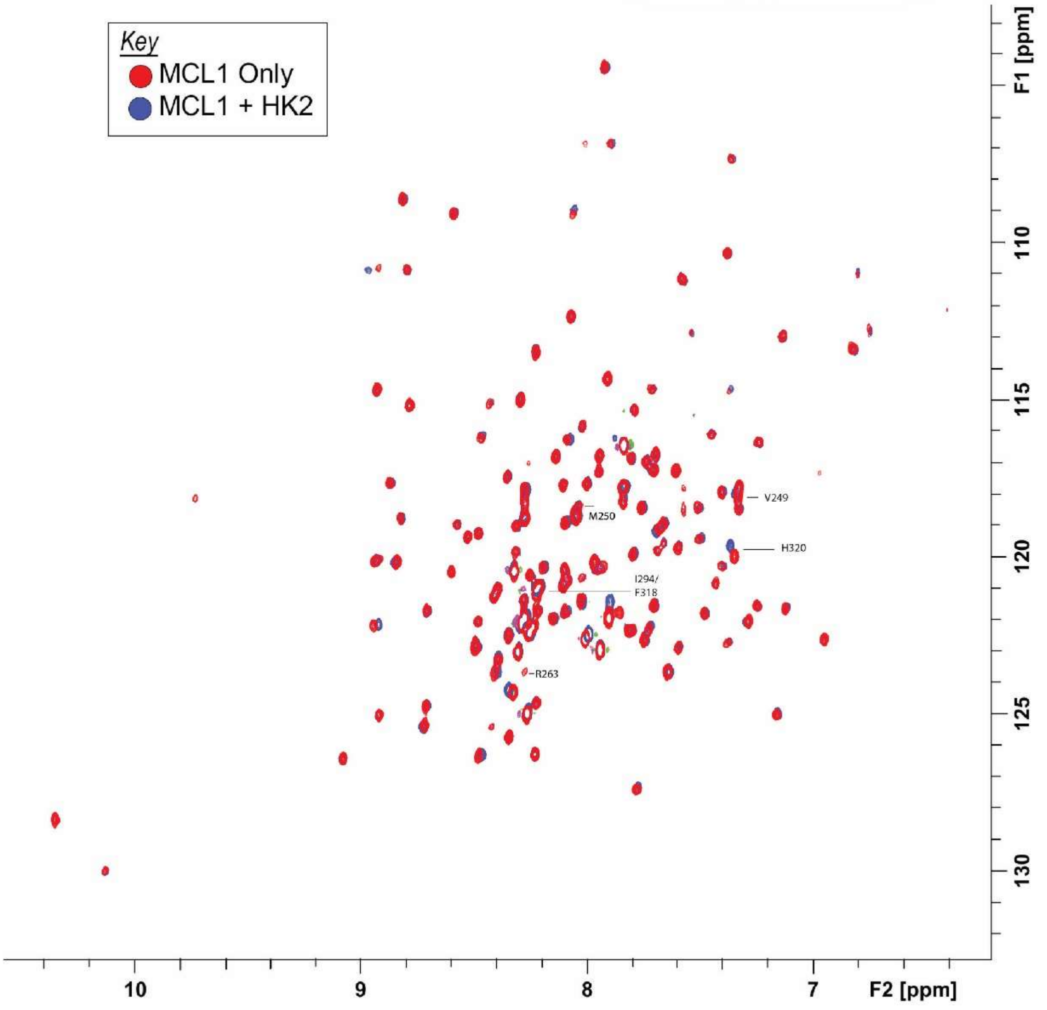

Supplemental Figure S5. Overlayed 2D [^{1}H-^{15}N]-TROSY HSQC of MCL1 and MCL1 + HK2.

Red spectra is 2D [^{1}H-^{15}N]-TROSY HSQC spectra of 35μM ^{15}N-MCL1 + 10% D_2O. Blue is 35μM ^{15}N-MCL1 + 38.5μM HK2 + 10% D_2O. Labels indicate select BH3-binding cleft residues.

A

	K_{Cat} (s^{-1})	$K_{Glucose}$ (mM)	K_{ATP} (mM)
50nM HK2 *only*	29.6 ± 1.5	0.23 ± 0.02	1.37 ± 0.16
500nM MCL1	42.2 ± 1.8	0.18 ± 0.04	1.19 ± 0.12
250nM MCL1	40.1 ± 2.0	0.21 ± 0.05	1.23 ± 0.15
100nM MCL1	32.6 ± 0.9	0.19 ± 0.03	1.06 ± 0.08
50nM MCL1	30.1 ± 0.9	0.20 ± 0.05	1.04 ± 0.08
500nM SUMO	34.4 ± 1.1	0.19 ± 0.04	1.25 ± 0.10
250nM SUMO	31.4 ± 0.8	0.21 ± 0.04	1.14 ± 0.07
100nM SUMO	32.1 ± 1.0	0.20 ± 0.05	1.09 ± 0.08
50nM SUMO	27.4 ± 0.8	0.19 ± 0.05	0.99 ± 0.07

All rows contain 50nM HK2

B

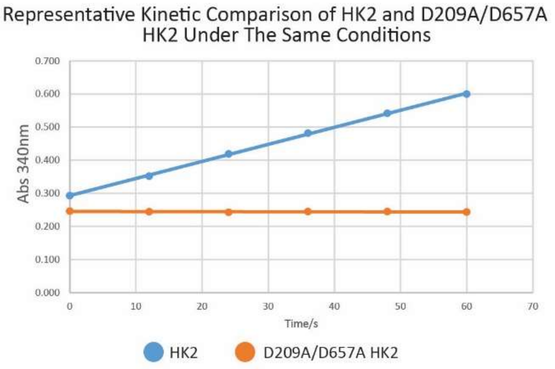

Supplemental Figure S6. Summary of Michaelis-Menten Kinetics for HK2.

A) Summary of Michaelis-Menten parameters obtained for HK2 only, HK2 + varying concentrations of MCL1 and HK2 + varying concentrations of SUMO. Each value and error were calculated from an averaged triplicate ATP and glucose Michaelis-Menten plots. B) Representative enzyme progress curves of HK2 or D209A/D657A HK2 collected at 4mM ATP + 1mM glucose demonstrating D209A/D657A HK2 lacks enzymatic activity.

Pathway	Metabolite	DMSO [μM/10^5 Cells]	S63845 [μM/10^5 Cells]	Percent Difference	p-value	Significance
Non-Essential Amino Acids	Alanine	1.41 ± 0.38	1.16 ± 0.32	-17.84%	0.429	ns
	Aspartic Acid	10.17 ± 1.53	5.73 ± 2.21	-43.68%	0.046	*
	Glycine	27.00 ± 4.91	22.74 ± 4.26	-15.78%	0.319	ns
	Glutamic Acid	50.74 ± 9.15	46.19 ± 4.03	-8.97%	0.475	ns
	Glutamine	75.00 ± 14.80	83.15 ± 10.81	10.86%	0.484	ns
	Serine	15.64 ± 4.57	18.65 ± 6.74	19.20%	0.558	ns
Non-Glucose Derived Metabolites	Isoleucine	22.17 ± 7.16	22.53 ± 4.82	1.63%	0.946	ns
	Leucine	21.23 ± 7.40	19.92 ± 4.06	-6.19%	0.801	ns
	Phenylalanine	7.15 ± 3.03	6.89 ± 1.45	-3.75%	0.897	ns

Supplemental Table S3. ^{1}H-NMR Metabolites from Non-Essential and Other Non-Glucose Derived Metabolites.

Glucose derived and non-glucose derived metabolite abundance with 5μM S63845 treatment. On day 2 cells were given fresh media and treated with 5μM S63845 or vehicular control and grown for 24hr. Metabolites were extracted, and ^{1}H-NMR was collected in 100mM Na_2HPO_4 + 0.02% NaN_3 + 500μM DSS in D_2O. Metabolites were quantified in Chenomx. Metabolite concentrations were normalized to viable cell count. Error was calculated for biological triplicate.

A

Pathway	Metabolite	DMSO [μM/10⁵ Cells]	S63845 [μM/10⁵ Cells]	Percent Difference	p-value	Significance
Glycolysis	Lactic Acid	6.10 ±1.29	3.47± 0.37	-43.17%	0.034	*
NAD+/NADH	NAD+	0.95 ± 0.04	0.93 ± 0.04	- 2.42%	0.482	ns
	NADH	1.19 ± 0.52	1.21 ± 0.32	1.97%	0.950	ns
TCA	Citric Acid	3.79 ± 0.42	2.34 ± 0.40	-38.25%	0.011	*
	Isocitrate	16.59 ± 1.41	11.61 ± 0.98	-30.03%	0.007	**
	Malic Acid	7.94 ± 2.57	8.52 ± 2.49	7.25%	0.794	ns
Ox-Phos	ADP	1.06 ± 0.53	1.27 ± 0.37	19.56%	0.610	ns
	ATP	2.36 ± 0.06	2.04 ± 0.36	-38.25%	0.005	**
PPP	NADP+	*Unquantifiable due to insufficient S/N*				
	NADPH					
Non-Essential Amino Acids	Alanine	1.02 ± 0.17	0.87 ± 0.27	-14.41%	0.474	ns
	Aspartic Acid	3.44 ± 0.96	2.89 ± 0.52	- 15.97%	0.433	ns
	Glycine	19.92 ± 1.97	18.85 ± 4.96	-5.34%	0.747	ns
	Serine	9.41 ± 1.32	9.34 ± 2.35	-0.73%	0.967	ns
Non-Glucose Derived Metabolites	Valine	13.43 ± 2.19	15.74 ± 4.05	17.20%	0.434	ns
	Tyrosine	4.30 ± 0.75	5.09 ± 1.01	18.34%	0.339	ns
	Methylmalonic Acid	4.67 ± 2.32	5.19 ± 1.68	11.13%	0.769	ns
	Phosphocholine	18.86 ± 0.69	15.83 ± 0.62	-16.07%	0.005	**

B

Pathway	Metabolite	DMSO Normalized A.U.C	S63845 Normalized A.U.C	Percent Difference	p-value	Significance
Glycolysis	Lactic Acid	1.04± 0.17	0.69± 0.12	-39.68%	0.047	*

Supplemental Figure S7. NCI-H23 Cell Metabolomics Reveal Similar Patterns of Metabolic Perturbation as H1299 Cells.

A) Glucose derived metabolite abundance changes with 3nM S63845 treatment in NCI-H23 cells. On day 2 cells were given fresh media and treated with 3nM S63845 or vehicular control and grown for 24hr. Metabolites were extracted, and ^{1}H-NMR was collected in 100mM Na_2HPO_4 + 0.02% NaN_3 + 500µM DSS in D_2O. Metabolites were quantified in Chenomx. $NADP^+$/NADPH levels could not be assessed due to signal loss upon S63845 treatment. Metabolite concentrations were normalized to viable cell count. B) ^{13}C lactic acid analysis in 3nM S63845 treatment in NCI-H23 cells. ^{13}C-NMR was collected in 100mM Na_2HPO_4 + 0.02% NaN_3 + 500µM DSS in D_2O. Concentration of lactic acid was quantified using area under the curve (A.U.C) of ^{13}C peak intensity measurements as calculated in Bruker Topspin. A.U.C was normalized to viable cell count. Other H1299 surveyed ^{13}C labelled metabolites could not be assessed in NCI-H23 due to signal loss upon S63845 treatment. Mean and error were calculated for biological triplicate.

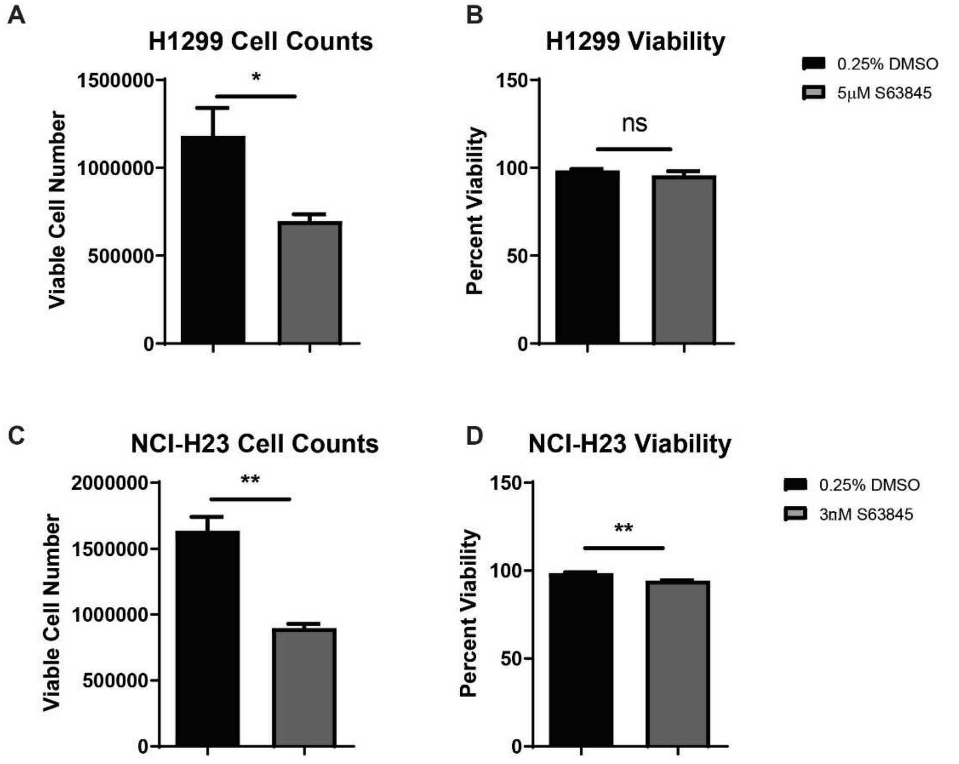

Supplemental Figure S8. Cell Counts and Viability of Cells Used for Metabolomics Analysis.

A-B) Cell counts of H1299 cells collected from matched surrogate samples. Viability of H1299 cells as assessed by trypan blue. C-D) A-B) Cell counts of NCI-H23 cells collected from matched surrogate samples. Viability of NCI-H23 cells as assessed by trypan blue. Error bars were calculated for biological triplicate.

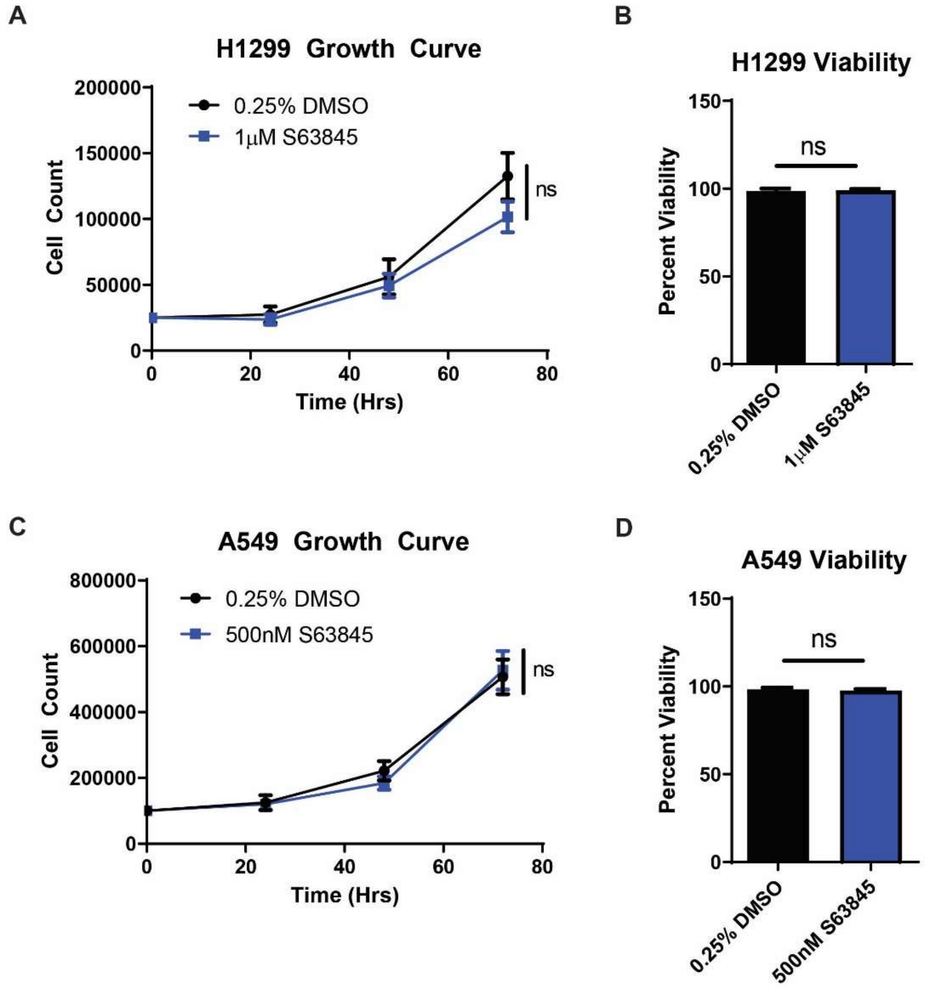

Supplemental Figure S9. S63845 Does Not Significantly Impact Proliferation Without Glucose Deprivation.

A) Proliferation curves of H1299 cells treated with vehicular control or 1μM MCL1 inhibitor S63845. B) Viability and cell count of H1299 cells on day 3 after treatment as assessed by trypan blue. C) Proliferation curves of A549 cells treated with vehicular control or 500nM MCL1 inhibitor S63845. D) Viability and cell count of A549 cells on day 3 after treatment as assessed by trypan blue. Error bars were calculated for biological triplicate.

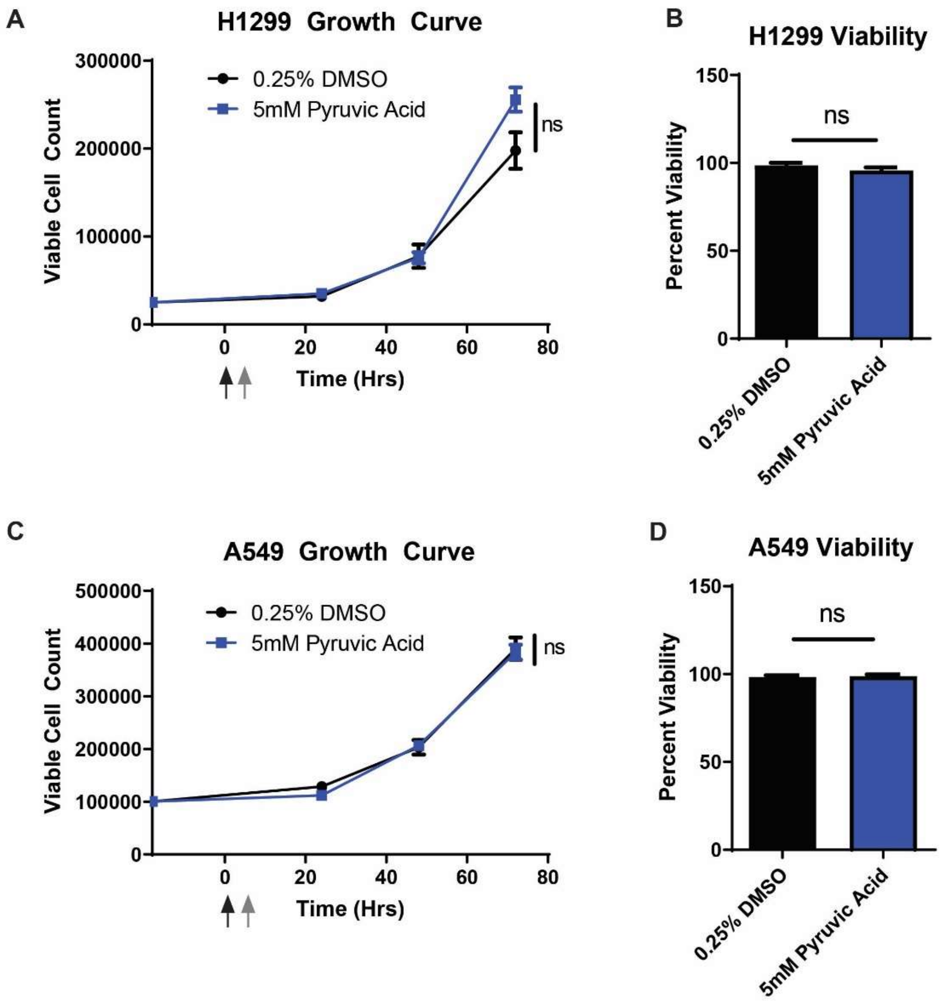

Supplemental Figure S10. 5mM Pyruvic Acid Treatment Does Not Significantly Impact Proliferation.

A) Proliferation curves of H1299 cells treated with vehicular control or vehicular control in media supplemented with 5mM pyruvic acid. Black arrow represents the start of glucose depletion to simulate glucose metabolic stress (6hrs). Grey arrow represents reintroduction of glucose as well as treatment with indicated group. B) Viability and cell count of H1299 cells on day 3 after treatment as assessed by trypan blue. C) Proliferation curves of A549 cells treated with vehicular control or vehicular control in media supplemented with 5mM pyruvic acid. Black arrow represents the start of glucose depletion to simulate glucose metabolic stress (6hrs). Grey arrow represents reintroduction of glucose as well as treatment with indicated group. D) Viability and cell count of A549 cells on day 3 after treatment as assessed by trypan blue. Error bars were calculated for biological triplicate
